# Mispositioned Hydrus Microstents: A Case Series Imaged with NIDEK GS-1 Gonioscope

**DOI:** 10.1155/2022/1605195

**Published:** 2022-09-08

**Authors:** Daniel Laroche, Alexander Martin, Aaron Brown, Sohail Sakkari, Chester Ng

**Affiliations:** ^1^New York Eye and Ear Institute of Mount Sinai, New York, NY, USA; ^2^Advanced Eyecare of New York, New York, NY, USA; ^3^Donald and Barbara, Zucker School of Medicine at Hofstra/Northwell, Hempstead, NY, USA

## Abstract

*Introduction*. The Hydrus microstent has become a common procedure in glaucoma surgery intended to improve outcomes of cataract surgery to lower intraocular pressure. Despite proper placement, this device can subsequently be noted to not be in the proper position. In this case series, we report mispositioned Hydrus microstents in five different patients and use NIDEK GS-1 gonioscopy. *Case Report*. We report five different patients who had cataract surgery and who were subsequently noted to have mispositioned Hydrus stents. No stents needed to be removed. All patients had improved vision and stable visual fields, and none required secondary surgery. *Management and Outcome*. In each case of mispositioned stents, vision was significantly improved and there was no inflammation or other complications noted. *Discussion*. Hydrus stents can be noted to be mispositioned during the post-operative period after successful insertion. This can often be well tolerated requiring no further intervention.

## 1. Introduction

Glaucoma is a leading cause of irreversible blindness worldwide [1, 2]. The Hydrus stent combined with cataract surgery has been shown to reduce intraocular pressure and preserve visual field better than cataract surgery alone in patients with glaucoma [3]. We have identified some cases of Hydrus stents that were subsequently noted to be mispositioned post-operatively. Here we report the patient outcome in these cases and how the patients were managed.

Gonioscopy is an important part of glaucoma evaluation in an ophthalmic examination. The technique is most often used to determine whether a patient has open or closed anterior chamber iridocorneal angles (ICAs) while also allowing one to carefully study the ICA to observe its anatomy [4]. A new area of particular interest is the examination of microinvasive glaucoma surgery (MIGS) device positioning in patients who have had glaucoma surgery [5, 6]. This is essential for assessing the angle appearance, pre-operative angle surgery preparation, and assessing post-operative placement of MIGS device and surgical outcome. This is also important in assessing whether the device is properly positioned to effectively lower intraocular pressure (IOP) and whether there may be a higher risk of post-operative complications.

One specific MIGS device, the Hydrus stent, is a relatively new product that has been proven effective in patients due to its relatively larger size and its ability to scaffold Schlemm's canal to facilitate aqueous outflow without being easily obstructed by pigment [7]. However, because the stent is larger in size, its ideal surgical placement can be slightly more challenging than that of other alternatives, such as the iStent Inject [8] or other non-device techniques, such as goniotomy, for less-experienced surgeons. Having performed hundreds of Hydrus microstents with success, we have identified a few cases of mispositioned stents noted post-operatively. Here we report these cases to share and understand any associated consequences of slight mispositioning of the stent. Naturally, the post-surgical positioning can only be properly evaluated with gonioscopy.

While manual gonioscopy is useful for examining surgical devices, it is limited because it offers only a partial view and short window to evaluate a given MIGS device since the clinician can only assess the device for as long as a patient is able to cooperate during the procedure [9–11]. In these instances, automated gonioscopy with the GS-1 automated gonioscope is very helpful because of its 360-degree, high-quality image capturing ability [12]. This machine takes sixteen-angle GS-1 images of the angle and can create one complete circular image. This facilitates viewing both the proximal and distal end of the Hydrus stent positioning in glaucoma patients who have had cataract extraction with glaucoma surgery to determine whether mispositioning of the stent can lead to adverse events in such patients.

## 2. Methods

This is a retrospective case series based on glaucoma patients who have been evaluated and treated at Advanced Eye Care of New York. Medical records of patients who have had cataract extraction and Hydrus stent placement were reviewed. Patients who followed up and underwent gonioscopy and had a stent noted to be mispositioned were identified and imaged with GS-1 automated gonioscope. All patients provided written informed consent in accordance with the Declarations of Helsinki.

### 2.1. Case 1

**Figure 1:**
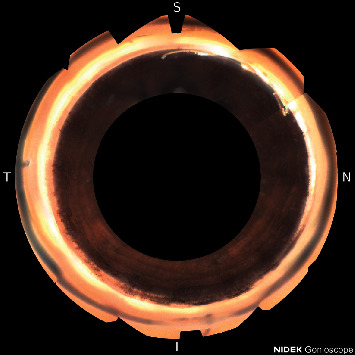
Extrusion of the distal tip of the Hydrus stent from Schlemm's canal.

This case was a 52-year-old black male with a history of primary open-angle glaucoma status-post (s/p) cataract extraction, Hydrus stent placement, and canaloplasty with OMNI surgical device in the right eye. Pre-operatively the patient had been on 5 medications in the right eye with IOP in the 20s. At the 9-month post-operative visit, the IOP was 10 mmHg with topical medications of timolol-brimonidine (Allergan) bid and dorzolamide 2% (Sandoz) tid. Axial length of the right eye was 24.72 mm. Imaging of the procedure with the GS-1 showed an extrusion of the distal tip of the Hydrus stent from Schlemm's canal, pictured in Figure 1. The patient had improvement of vision and visual field, and there was no inflammation or other complications.

### 2.2. Case 2

**Figure 2:**
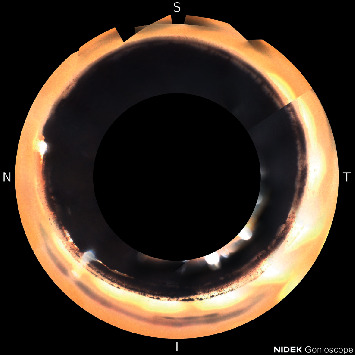
Distal extrusion of the Hydrus stent from Schlemm's canal.

This case was a 64-year-old black male with a cataract and mild stage primary open-angle glaucoma. He had been treated with timolol-brimonidine (Allergen) and travoprost (Alcon) in the left eye, with an average IOP of 10 mm·Hg pre-operatively. The patient agreed with written informed consent for cataract extraction, Hydrus stent placement, and OMNI canaloplasty in the left eye. Following surgery, BCVA improved to 20/20 at 6 months post op. IOP was 11 mm Hg on timolol-brimonidine and travoprost Z qpm. HVF 24-2 showed an improvement to −4.37 MD and 88% VFI. Axial length of the left eye was 23.56 mm. Imaging of the ICA showed distal extrusion of the Hydrus stent from Schlemm's canal, as seen in Figure 2. The patient had improvement of vision and visual field, and there was no inflammation or other complications.

### 2.3. Case 3

**Figure 3:**
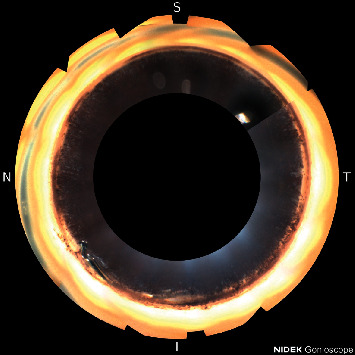
Hydrus stent distal tip mispositioned posterior to, rather than into, Schlemm's canal.

An 86-year-old black male with a history of mild stage pre-perimetric primary open-angle glaucoma had BCVA of 20/50, and he was treated with latanoprost qpm only. Average IOP was 11 mm·Hg on treatment. His CDR was 0.85, and average OCT-ONH RNFL thickness was 61 *μ*m. HVF 24-2 showed a MD of −4.98 and VFI of 98%. The patient provided written informed consent for cataract extraction and Hydrus stent placement in the left eye. Following surgery, BCVA improved to 20/20 12 months post op. IOP was 12 mm·Hg on no medications. HVF 24-2 showed a slight improvement to −4.07 MD with a stable 97% VFI. Axial length of the left eye was 29.12 mm. Imaging of the angle, as pictured in Figure 3, showed the Hydrus stent distal tip mispositioned posterior to, rather than into, Schlemm's canal. The patient had improvement of vision and visual field, and there was no inflammation or other complications.

### 2.4. Case 4

**Figure 4:**
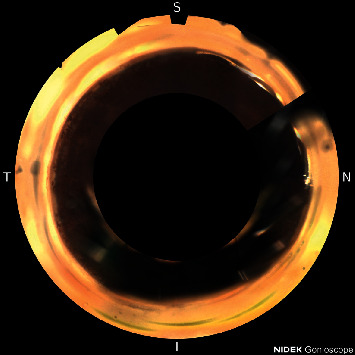
Extrusion of the distal tip of the Hydrus stent from Schlemm's canal.

A 68-year-old black female with a history of cataracts and moderate stage chronic angle-closure glaucoma had BCVA of 20/40 and was treated with latanoprost qpm, brimonidine 0.15% tid, and dorzolamide-timolol bid. Average IOP was 10 mm·Hg on medications. CDR was 0.75, and OCT-ONH showed an average RNFL thickness of 74 *μ*m. HVF 24-2 had a VFI of 76% and MD of −9.81. She agreed with written informed consent to have cataract extraction, Hydrus stent placement, and canaloplasty with OMNI surgical device in the right eye. Following surgery, the patient's BCVA improved to 20/25. IOP was 12 mmHg 7 months post op on no medications. HVF 24-2 showed an improved VFI of 81% and MD of −9.57 after surgery. Axial length of the right eye was 22.25 mm. Gonioscopy and imaging of the procedure with the GS-1 showed an extrusion of the distal tip of the Hydrus stent from Schlemm's canal, as seen in Figure 4. The patient had improvement of vision and visual field, and there was no inflammation or other complications.

### 2.5. Case 5

**Figure 5:**
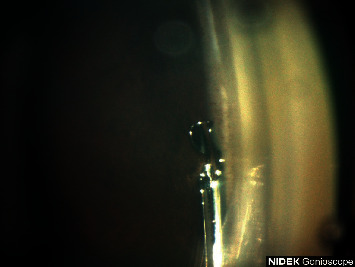
Hydrus stent not fully positioned and adjacent goniotomy seen performed by Sinskey hook.

A 65-year-old black male with a history of mild stage primary open-angle glaucoma underwent cataract extraction with Hydrus stent placement, OMNI canaloplasty, 21G MIMS, and goniotomy adjacent to the Hydrus stent. Before surgery, BCVA was 20/40, and the patient was treated with latanoprost qpm. Average IOP was 13 mm·Hg on medications. CDR was 0.85, and OCT-ONH RNFL thickness was 79 *μ*m. HVF 24-2 showed a MD of −10.89 and VFI of 71% prior to surgery. Following surgery, BCVA improved to 20/30 4 months post op. IOP was 14 mm·Hg without medications. HVF 24-2 was essentially not reliable with −13.83 MD and 73% VFI. Axial length of the left eye was 24.91 mm. GS-1 imaging showed incomplete insertion of the Hydrus stent with an adjacent goniotomy, as shown in Figure 5. The patient had improvement of vision, and there was no inflammation or other complications.

## 3. Discussion

Early cataract surgery with placement of a Hydrus stent in Schlemm's canal can help reduce IOP by increasing anterior chamber depth [13], opening the ICA [14], increasing aqueous outflow through the trabecular meshwork (TM), and creating a scaffolding to ensure access to collector channels [15, 16]. While this has been discussed in other studies, one relatively uncovered topic is whether a mispositioned stent has a significant effect on IOP lowering effectivity. Hydrus stent mispositioning has been described as an adverse event elsewhere [15], but long-term follow-up with photo documentation of such mispositioned devices has not been discussed. In this report, five cases of mispositioned Hydrus stents are discussed, and the results suggest that positioning may not necessarily negatively affect IOP. Also, we point out that with a Sinskey hook, you can make an additional 1-2 clock hour goniotomy in the opposite direction of the stent in case the distal tip is outside of the canal for the distal 1-2 clock hours, as imaged in Figure 5. Studies have shown that with 3 clock hours of stenting the canal, you can get the greatest reduction of IOP [7].

In all cases of the mispositioned Hydrus stents, surgery in conjunction with cataract surgery resulted in an improvement of BCVA by at least two lines and improvement or stability in HVF defects. This was likely due to the removal of a visually significant cataract in each case. Average IOP before surgery was 12.8 mm·Hg, while average IOP after surgery was 11.8 mm Hg—an 8% decrease. The IOP reduction in these cases is very small since we did not perform a pre-operative washout of topical glaucoma medications as is often done in clinical trials. Other clinical trials show a roughly 20% reduction of IOP [15, 17, 18]. Additionally, medication usage from before to after surgery decreased from an average of 2.8 to 1.2, respectively—a 58% decrease. In each case, IOP was still at target after surgery, with the added benefit of the patient most likely being able to use 1 to 2 less medications than before surgery. As a result, the patient's quality of life was better off, and compliance was likely to be improved due to reduced medication burden [19].

In addition to the above, one other benefit of the earlier surgery in each case is that the underlying mechanism contributing to glaucoma was addressed. As the cataract matures, it increases in thickness, causing pigment liberation, leading to TM obstruction and narrowing of the ICA [20, 21]. This will only worsen over time as the cataract continues to mature, resulting in increased IOP. Removal of the cataract and bypassing the TM with a stent address all these concerns, leading to better long-term health of the TM and reducing the risk of glaucoma progression over time. Additionally, as seen in the final case presented above, if the surgeon is uncertain whether the stent was placed ideally, adjacent goniotomy with a Sinskey hook can be used as insurance to help maintain lower IOP.

These generally positive results aside, one question remains: what causes the Hydrus stent to be improperly positioned in Schlemm's canal? The stent has a pre-set curvature at the time of production. The curve of the stent is that of a 12 mm diameter circle. The average corneal diameter is 11.74 mm. The amount of curvature is retained at body temperature. This is the “shape memory” property of Nitinol [22]. Left unconstrained by its surroundings, it is supposed to return to the factory setting. However, in the inserter, the stent has a greater curvature to facilitate insertion into Schlemm's canal. This may affect the memory of the device. Nonetheless, the device is very flexible and is often constrained within the confines of the trabecular meshwork and Schlemm's canal in most cases. It is possible that in eyes with greater axial length, there may be a larger arc than the stent's flex is designed to accommodate for, causing the distal tip to come back through the trabecular meshwork. The average axial length of the eyes discussed in these cases is 24.93 mm. The average axial length in adults varies from 22 to 25 mm depending on refractive error [23]. Nearly all eyes discussed are under 25 mm in length, with the one exception being over 29 mm in length, suggesting that long axial length creating a larger arc was not a likely cause for stent mispositioning in most cases. However, this does not necessarily mean that the arc of the angle did not play a role in the stent mispositioning, and future work should be done to examine the relationship between anterior segment arc and stent mispositioning.

Another potential explanation for stent mispositioning is the presence of possible adhesions or herniations of Schlemm's canal that led to a discontinuity during insertion of the stent, deflecting the tip towards the anterior chamber. This can potentially reduce the efficacy of the Hydrus stent if there is less extensive scaffolding of Schlemm's canal. Adding additional 1-2 clock hour adjacent goniotomy with a Sinskey hook in the opposite direction of insertion can potentially aid in ensuring the success of the stent if the distal end becomes mispositioned over time. The thickness of Schlemm's canal may vary with its strength to fully hold the stent as well. There could be variation in anatomy and effects of integrity from previous interventions such as laser treatments. This should also be further investigated.

While the outcomes discussed here are favorable even with stents that are not positioned ideally, further work needs to be done on understanding the long-term consequences of having a mispositioned Hydrus stent in Schlemm's canal. The longest follow-up length discussed here is 19 months, which is still relatively short-term. In addition, results were skewed by the first case in this report, as the IOP change was much more significant compared to the subsequent cases. Furthermore, a large-scale study should be done to observe true, statistically significant results. None of the stents needed to be removed or repositioned. There were no adverse effects of cystoid macular edema or chronic inflammation noted.

## 4. Conclusion

Hydrus stents may potentially be mispositioned distally, which can be noted during the post-operative period. These cases were able to be imaged well with the NIDEK GS-1 gonioscope. While the Hydrus stent can be challenging to position ideally, this study suggests that even with an imperfect placement, the stent can be highly effective and can improve the quality of life and long-term ocular health for patients. Furthermore, adjacent Sinskey hook goniotomy can be used to help enhance the effect of the misplaced stent, if needed. Thus, even less-experienced surgeons need not be hesitant to use this device as a treatment option in their surgical management of glaucoma patients.

## Data Availability

The stent placement image data used to support the findings of this study are available from the corresponding author upon request.

## References

[1] Parihar J. K. S. (2016). Glaucoma: the “Black hole” of irreversible blindness. *Medical Journal Armed Forces India*.

[2] Steinmetz J. D., Bourne R. R. A., Briant P. S. (2021). Causes of blindness and vision impairment in 2020 and trends over 30 years, and prevalence of avoidable blindness in relation to VISION 2020: the Right to Sight: an analysis for the Global Burden of Disease Study. *Lancet Global Health*.

[3] Otarola F., Virgili G., Shah A., Hu K., Bunce C., Gazzard G. (2020). Ab interno trabecular bypass surgery with Schlemm’s canal microstent (Hydrus) for open angle glaucoma. *Cochrane Database of Systematic Reviews*.

[4] Gedde S. J., Vinod K., Wright M. M. (2021). Primary open-angle glaucoma preferred practice pattern. *Ophthalmology*.

[5] Lavia C., Dallorto L., Maule M., Ceccarelli M., Fea A. M. (2017). Minimally-invasive glaucoma surgeries (MIGS) for open angle glaucoma: a systematic review and meta-analysis. *PLoS One*.

[6] Cutolo C. A., Bonzano C., Scotto R. (2021). Moving beyond the slit-lamp gonioscopy: challenges and future opportunities. *Diagnostics*.

[7] Ahmed I. I. K., Fea A., Au L. (2020). A prospective randomized trial comparing Hydrus and iStent microinvasive glaucoma surgery implants for standalone treatment of open-angle glaucoma: the COMPARE study. *Ophthalmology*.

[8] Ziaei H., Au L. (2021). Manchester iStent study: long-term 7-year outcomes. *Eye*.

[9] Aung T., Quek D. L., Nongpiur M., Perera S. (2011). Angle imaging: advances and challenges. *Indian Journal of Ophthalmology*.

[10] Hertzog L. H., Albrecht K. G., LaBree L., Lee P. P. (1996). Glaucoma Care and conformance with preferred practice patterns. *Ophthalmology*.

[11] Teixeira F., Sousa D. C., Leal I., Barata A., Neves C. M., Pinto L. A. (2020). Automated gonioscopy photography for iridocorneal angle grading. *European Journal of Ophthalmology*.

[12] Riva I., Micheletti E., Oddone F. (2020). Anterior chamber angle Assessment techniques: a review. *Journal of Clinical Medicine*.

[13] Ning X., Yang Y., Yan H., Zhang J. (2019). Anterior chamber depth-a predictor of refractive outcomes after age-related cataract surgery. *BMC Ophthalmology*.

[14] Grehn F., Klink T. (2020). Management of glaucoma and cataract. *Albert and Jakobiec’s Principles and Practice of Ophthalmology*.

[15] Samuelson T. W., Chang D. F., Marquis R. (2019). A Schlemm canal microstent for intraocular pressure reduction in primary open-angle glaucoma and cataract: the HORIZON study. *Ophthalmology*.

[16] Camras L. J., Yuan F., Fan S. (2012). A novel schlemm’s canal scaffold increases outflow facility in a human anterior segment perfusion model. *Investigative Opthalmology & Visual Science*.

[17] Pfeiffer N., Garcia-Feijoo J., Martinez-de-la-Casa J. M. (2015). A randomized trial of a schlemm’s canal microstent with phacoemulsification for reducing intraocular pressure in open-angle glaucoma. *Ophthalmology*.

[18] Fea A. M., Rekas M., Au L. (2017). Evaluation of a schlemm canal scaffold microstent combined with phacoemulsification in routine clinical practice: two-year multicenter study. *Journal of Cataract & Refractive Surgery*.

[19] Lemij H. G., Hoevenaars J. G., van der Windt C., Baudouin C. (2015). Patient satisfaction with glaucoma therapy: reality or myth?. *Clinical Ophthalmology*.

[20] Laroche D., Capellan P. (2021). The aging lens and glaucoma in persons over 50: why early cataract surgery/refractive lensectomy and microinvasive trabecular bypass can prevent blindness and cure elevated eye pressure. *Journal of the National Medical Association*.

[21] Altan C., Bayraktar S., Altan T., Eren H., Yilmaz O. F. (2004). Anterior chamber depth, iridocorneal angle width, and intraocular pressure changes after uneventful phacoemulsification in eyes without glaucoma and with open iridocorneal angles. *Journal of Cataract & Refractive Surgery*.

[22] Chen G., Liu J., Dong Z. (2021). Understanding mechanisms of shape memory function deterioration for nitinol alloy during non-equilibrium solidification by electron beam. *Journal of Advanced Research*.

[23] Bhardwaj V., Rajeshbhai G. P. (2013). Axial length, anterior chamber depth-a study in different age groups and refractive errors. *Journal of Clinical and Diagnostic Research*.

